# Supplementary material for: Maternal Supplementation with a Cocoa Extract during Lactation Deeply Modulates Dams’ Metabolism, Increases Adiponectin Circulating Levels and Improves the Inflammatory Profile in Obese Rat Offspring
Source: Nutrients. 2022 Dec 3;14(23):5134. doi: 10.3390/nu14235134 (PMC9738144; doi:10.3390/nu14235134)
Supplement: Supplementary file 1 [file nutrients-14-05134-s001.zip › Supplementary_Table_S1.pdf]

Table S1. Nucleotide sequences of primers used for real-time quantitative PCR.

Gene	Forward primer (5′ to 3′)	Reverse primer (5′ to 3′)
Acc1	TGCAGGTATCCCCACTCTTC	TTCTGATTCCCTTCCCTCCT
Acc2	TCCTTTCAGACCTCCTCTCG	GATAACCCTGTTGCCTCCAA
Adipoq	GTTCCAGGACTCAGGATGCT	CGTCTCCCTTCTCTCCCTTC
Adipor1	TCTCCATCGTCTGTGTCCTG	AATCCGAGCAGCATAAAGGC
Adipor2	AGCCATTCTCTGCCTTTCCT	ACATGTCCCACTGAGAGACG
Atgl	CACTTTAGCTCCAAGGATGA	TGGTTCAGTAGGCCATTCCT
β-actin	TACAGCTTCACCACCACAGC	TCTCCAGGGAGGAAGAGGAT
Cd36	GTCCTGGCTGTGTTTGGA	GCTCAAAGATGGCTCCATTG
Cd74	CCACCTAAAGAGCCACTGGA	AGGGACGGTGAAGCAGATAC
Cpt1α	GCTCGCACATTACAAGGACAT	TGGACACCACATAGAGGCAG
Cpt1β	GCAAACTGGACCGAGAAGAG	CCTTGAAGAAGCGACCTTTG
Dgat1	CAGACAGCGGTTTCAGCAAT	AGGGGTCCTTCAGAAACAGAG
DsbA-L	GCTTCACGTTCGCTTCTCTC	GCCGCAACTTCAGCTTGATA
Emr1	GATGTGGAGGATGGGAGATG	GCACGAAACAACAGGAAGGT
Ero1-Lα	TGTCAAACCCTGCCATTCTG	TCCACATACTCAGCATCGGG
Fas	CGGCGAGTCTATGCCACTAT	ACACAGGGACCGAGTAATGC
Gpat	CAGCGTGATTGCTACCTGAA	CTCTCCGTCCTGGTGAGAAG
Had	ATCGTGAACCGTCTCTTGGT	AGGACTGGGCTGAAATAAGG
Hprt	TCCCAGCGTCGTGATTAGTGA	CCTTCATGACATCTCGAGCAAG
Hsl	TCACGCTACATAAAGGCTGCT	CCACCCGTAAAGAGGGAACT
Il10	ACCTGGTAGAAGTGATGCCC	GCTCCACTGCCTTGCTTTTA
iNOS	GGCAGCTACTGGGTCAAAGA	TCTGAGGGCTGACACAAGG
Mcp1	AGGCAGATGCAGTTAATGCCC	ACACCTGCTGCTGGTGATTCTC
Mgl1	CTCCAACACCAAGGCTGAAC	GGTCTTCAAGTCCTTCCCCA
Mrc1	CTGCAAGGAAGGTTGGCATT	CAGGCGTTGAAAGTGGAGTC
Pparα	GTGGCTGCTATAATTTGCTGTG	AGCTTCGGGAAGAGAAAGGTAT
Ppia	CCAAACACAAATGGTTCCCAGT	ATTCCTGGACCCAAAACGCT
Tfrc	ATCATCAAGCAGCTGAGCCAG	CTCGCCAGACTTTGCTGAATTT
Tnfα	TGCCTCAGCCTCTTCTCATT	GCTTGGTGGTTTGCTACGAC

Primer pairs for PCR were designed using Primer3 software, and the sequence information was obtained from GenBank. *Acc1*, acetyl CoA carboxylase 1; *Acc2*, acetyl CoA carboxylase 2; *Adipoq*, adiponectin; *Adipor1*, adiponectin receptor 1; *Adipor2*, adiponectin receptor 2; *Atgl*, adipose triglyceride lipase; *β-actin*, actin beta; *Cd36*, fatty acid translocase, homologue of CD36; *Cd74*, major histocompatibility complex, class II invariant chain; *Cpt1α*, carnitine palmitoyltransferase 1 alpha; *Cpt1β*, carnitine palmitoyltransferase 1 beta; *Dgat1*, diacylglycerol acyltransferase 1; *DsbA-L*, disulfide-bond-A oxidoreductase-like protein; *Emr1*, EGF-like module containing, mucin-like, hormone receptor-like 1; *Ero1-Lα*, endoplasmic reticulum oxidoreductin 1-like protein alpha; *Fas*, fatty acid synthase; *Gpat*, glycerol-3-phosphate acyltransferase; *Had*, hydroxyacyl-CoA dehydrogenase; *Hprt*, hypoxanthine guanine phosphoribosyl transferase; *Hsl*, hormone-sensitive lipase; *Il10*, interleukin 10; *iNOS*, nitric oxide synthase 2, inducible; *Mcp-1*, monocyte chemoattractant protein-1; *Mgl1*, C-type lectin domain family 10, member A; *Mrc1*, mannose receptor, C type 1; *Pparα*, peroxisome proliferator-activated receptor alpha; *Ppia*, peptidylprolyl isomerase A; *Tfrc*, transferrin receptor; *Tnfα*, tumor necrosis factor.
