# Supplementary material for: Maternal Supplementation with a Cocoa Extract during Lactation Deeply Modulates Dams’ Metabolism, Increases Adiponectin Circulating Levels and Improves the Inflammatory Profile in Obese Rat Offspring
Source: Nutrients. 2022 Dec 3;14(23):5134. doi: 10.3390/nu14235134 (PMC9738144; doi:10.3390/nu14235134)
Supplement: Supplementary file 1 [file nutrients-14-05134-s001.zip › Supplementary_Table_S2.pdf]

Table S2. Lipid metabolism-related parameters analyzed in dams' mammary gland by Nuclear Magnetic Resonance.

μmols /g mammary gland	**VEH**	**CCX**
Free Cholesterol	0.059 ± 0.002	0.060 ± 0.003
Esterified Cholesterol	0.004 ± 0	0.004 ± 0
Total Cholesterol	0.063 ± 0.002	0.064 ± 0.003
Triglycerides	1.04 ± 0.09	0.82 ± 0.04 *
Diglycerides	0.024 ± 0.002	0.023 ± 0.001
Monoglycerides	0.0005 ± 0.0001	0.0006 ± 0.0001
Phosphatidyl Choline	0.17 ± 0.01	0.17 ± 0.01
Phosphatidyl Ethanolamine	0.080 ± 0.004	0.080 ± 0.004
Phosphatidyl Inositol	0.006 ± 0	0.005 ± 0
Sphingomyelin	0.015 ± 0.001	0.014 ± 0.001
Plasmalogen	0.017 ± 0.001	0.013 ± 0.002
Omega-3	0.069 ± 0.004	0.057 ± 0.003 *
ARA+EPA	0.088 ± 0.005	0.089 ± 0.003
Oleic acid	0.72 ± 0.06	0.53 ± 0.03 *
DHA	0.008 ± 0.001	0.006 ± 0 *
Linoleic acid	1.12 ± 0.09	0.92 ± 0.04
PUFAs (%)	12.3 ± 0.9	13.6 ± 0.6
MUFAs (%)	49.1 ± 1.9	50.8 ± 1.6

Dams were fed with chow and received a daily oral dose of CCX (200 mg per kg body weight) (CCX group) or the vehicle (VEH group) during lactation (21 days). CCX, cocoanox extract; ARA+EPA, arachidonic + eicosapentaenoic acid; DHA, docosahexaenoic acid; PUFAs, polyunsaturated fatty acids; MUFAs, monounsaturated fatty acids. Data are the mean ± SEM (n=8). * $p<0.05$ vs VEH group (Student's t-test).
